# A tailored polylactic acid/polycaprolactone biodegradable and bioactive 3D porous scaffold containing gelatin nanofibers and Taurine for bone regeneration

**DOI:** 10.1038/s41598-020-70155-2

**Published:** 2020-08-07

**Authors:** Hadi Samadian, Saeed Farzamfar, Ahmad Vaez, Arian Ehterami, Arindam Bit, Mostafa Alam, Arash Goodarzi, Gholamhossein Darya, Majid Salehi

**Affiliations:** 1 Nano Drug Delivery Research Center, Health Technology Institute, Kermanshah University of Medical Sciences, Kermanshah, Iran; 2 Department of Tissue Engineering and Applied Cell Sciences, School of Advanced Technologies in Medical Sciences and Technologies, Tehran University of Medical Sciences, Tehran, Iran; 3 Department of Tissue Engineering and Applied Cell Sciences, School of Advanced Medical Sciences and Technologies, Shiraz University of Medical Sciences, Shiraz, Iran; 4 Department of Mechanical Engineering, Science and Research Branch, Islamic Azad University, Tehran, Iran; 5 National Institute of Technology, Raipur, India; 6 Department of Oral and Maxillofacial Surgery, School of Dentistry, Shahid Beheshti University of Medical Sciences, Tehran, Iran; 7 Department of Tissue Engineering, School of Advanced Technologies in Medicine, Fasa University of Medical Sciences, Fasa, Iran; 8 Department of Comparative Biomedical Science, School of Advanced Medical Sciences and Technologies, Shiraz University of Medical Sciences, Shiraz, Iran; 9 Department of Tissue Engineering, School of Medicine, Shahroud University of Medical Sciences, Shahroud, Iran; 10 Tissue Engineering and Stem Cells Research Center, Shahroud University of Medical Sciences, Shahroud, Iran

**Keywords:** Biomaterials, Tissues

## Abstract

The focus of the current study was to develop a functional and bioactive scaffold through the combination of 3D polylactic acid (PLA)/polycaprolactone (PCL) with gelatin nanofibers (GNFs) and Taurine (Tau) for bone defect regeneration. GNFs were fabricated via electrospinning dispersed in PLA/PCL polymer solution, Tau with different concentrations was added, and the polymer solution converted into a 3D and porous scaffold via the thermally-induced phase separation technique. The characterization results showed that the scaffolds have interconnected pores with the porosity of up to 90%. Moreover, Tau increased the wettability and weight loss rate, while compromised the compressive strengths. The scaffolds were hemo- and cytocompatible and supported cell viability and proliferation. The in vivo studies showed that the defects treated with scaffolds filled with new bone. The computed tomography (CT) imaging and histopathological observation revealed that the PLA/PCL/Gel/Tau 10% provided the highest new bone formation, angiogenesis, and woven bone among the treatment groups. Our finding illustrated that the fabricated scaffold was able to regenerate bone within the defect and can be considered as the effective scaffold for bone tissue engineering application.

## Introduction

Bone is a typical complex tissue with a hierarchical structure that supports the body, protects internal organs, facilitates movement, stores, and releases minerals. Bone defects are a serious illness that resulted in post-trauma, osteoporosis, congenital defects, arthritis, and neoplasm, etc*.* Due to their osteoinductivity and osteoconductivity, bone graft materials, as autografts, allografts, and xenografts have been used for bone defects and fractures treatment. Although they have some advantages, they suffer from the risk of disease transfer, an increase of operative time and cost, possible immunogenicity, and chronic pain^1–4^.

Remarkable attention has been laid toward bone tissue engineering, synthetic and natural biomaterials by orthopedics and researchers to overcome the drawbacks of the existing treatment regimes. Bone tissue engineering as an alternative to the current treatment approaches is the combination of biomaterials as tissue scaffolds and drug delivery vehicles, cell-stimulating agents, and bone lying cells^5,6^. The most important part of the bone tissue engineering is the scaffold which requires precise designing and processing to mimic the biological, structural, and mechanical properties of natural bone tissue. Inspired by the nature of bone, a proper bone formation and ingrowth requires a 3D, porous, structurally hierarchical nanocomposites and constructs that possess several levels of the organization, i.e., from the molecular arrangement of proteins up to the macroscopic tissue arrangement^7^.

Electrospun nanofibers have gained a great deal of attention as the bone tissue engineering scaffolds due to their promising properties, such as a resemblance to the extracellular matrix (ECM), high surface to volume ratio, flexibility in the fabrication process, and low-cost production^8,9^. A wide range of polymers and biopolymers have been electrospun and applied as the bone tissue engineering scaffolds^10^. Gelatin is a promising biopolymer derived from collagen and has been widely utilized for bone tissue engineering^11,12^. Lee et al. and Zha et al. showed that the incorporation of gelatin into the scaffolds promotes osteoblast cell attachment and proliferation^13,14^. Despite their wide applications in the bone tissue engineering, their translation to the clinics has not been achieved due to their 2D structure. Accordingly, electrospun nanofibers can be incorporated into the 3D structures as the nanocomposite filler^15^.

The thermally-induced phase separation (TIPS) technique is a valuable method to fabricate 3D scaffolds with adjustable porosity and interconnected pores, critical for bone tissue engineering. In this approach, the intended homogenous polymer solution underwents a phase separation under the proper temperature and separates into polymer-rich and polymer-lean regions. In the final step and after the solvent extraction step, the 3D porous scaffold is obtained^16^. In addition to the structural support, the bioactive molecules are also required for an effective and facilitated bone healing process. Various types of bioactive substances have been evaluated for bone tissue engineering, such as peptides, mineral crystals, and amino acids. Taurine (Tau) is an essential amino acid which its positive effects on bone metabolism and formation have been reported^17–19^. It is a sulfurated β-amino acid that is found in free form in animal sources^20,21^. It is documented that Tau is involved in calcium modulation, osmoregulation, antioxidation, membrane stabilization, neuromodulation, protein phosphorylation regulation^19,22,23^. Accordingly, in the current study, we aimed to combine the positive biological activities of electrospun (GNFs) and Tau with the structural features of the TIP-based scaffold to develop an innovative tissue engineering approach for bone regeneration.

## Results and discussions

### The surface morphology

**Figure 1:**
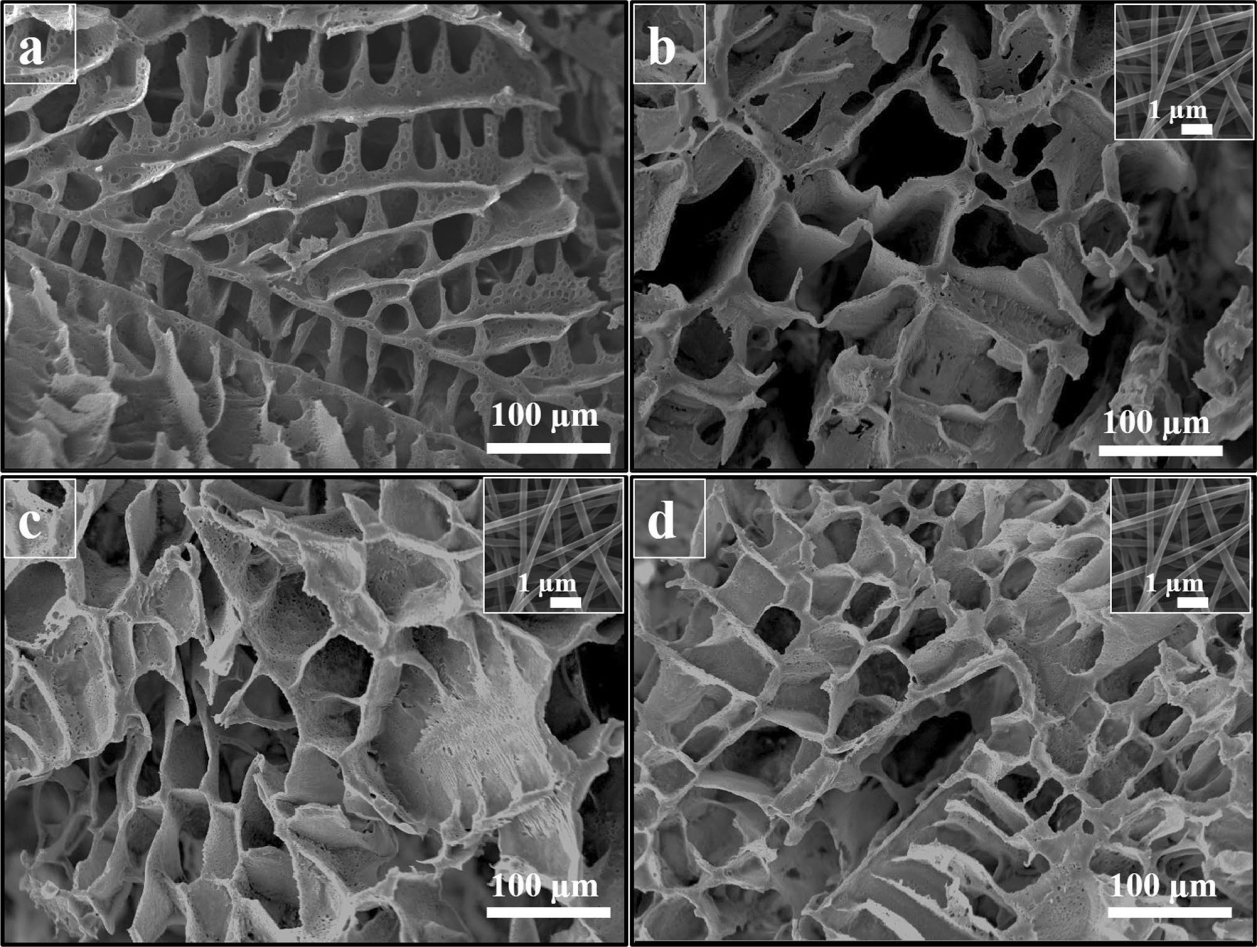
SEM micrograph of the prepared scaffolds. (**a**) PCL/PLA/GNF, (**b**) PCL/PLA/GNF/Tau 0.1%, (**c**) PCL/PLA/GNF/Tau 1%, and (**d**) PCL/PLA/GNF/ Tau 10%. The insets show SEM micrograph of GNFs.

SEM was used to evaluate the surface morphology of the cross-sectioned scaffolds, and the results are shown in Fig. 1. The image analysis showed that the fabricated GNFs have average diameter around 194 nm. The results of hydrogel imaging showed that the scaffolds have interconnected pore microstructure, which is critical for cell infiltration, nutrient, and waste transfer.

The pore size measurement using the ImageJ software (version 1.43, https://imagej.nih.gov/ij/, National Institutes of Health, Bethesda, Maryland) showed that the incorporating 0.1% Tau increased the pore size of PCL/PLA/GNF hydrogel from 37 ± 12 to 64 ± 11 µm. On the other hand, further elevating the concentration of Tau from 0.1 to 10% did not significantly influence the pore size (p < 0.3) and the connection between the pores.

### Wettability measurement

**Figure 2:**
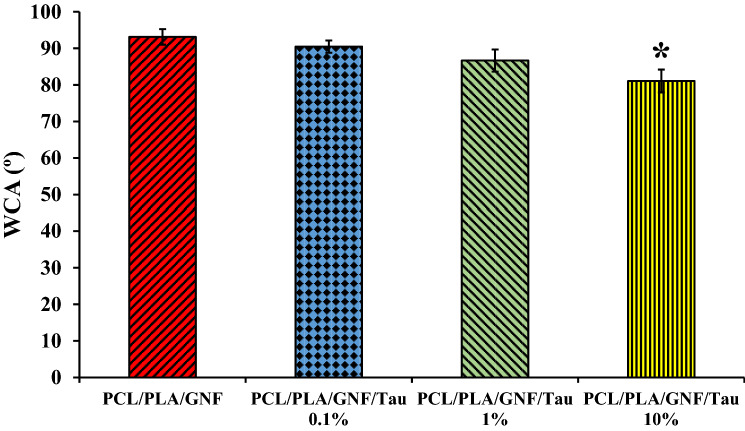
The water contact angle value of the prepared scaffolds. *Significance against PCL/PLA/GNF and PCL/PLA/GNF Tau 0.1% (p < 0.05).

The wettability is a critical property for scaffolds that determines the interactions of cells and biological fluid proteins with the scaffold^24,25^. Moreover, it impacts the degradation rate of a scaffold in the body^26,27^. The wettability of the prepared scaffold was evaluated with the water contact angle measurement method, and the results are presented in Fig. 2.

The results showed that there is an indirect correlation between the concentration of the Tau and the wettability of the scaffolds. Increasing the concentration of Tau to the scaffolds decreased the water contact angle value due to its hydrophilic nature. The influence of the Tau on the hydrophobicity of the scaffolds was statistically significant in 10% Tau group, which decreased the WCA from 108.9 ± 3.4 for PCL/PLA/GNF to 81.0 ± 3.1 for PCL/PLA/GNF/10% Tau scaffolds. Our previous study also showed that the addition of Tau makes the fabricated dressing more hydrophilic^28^.

### Weight loss

**Figure 3:**
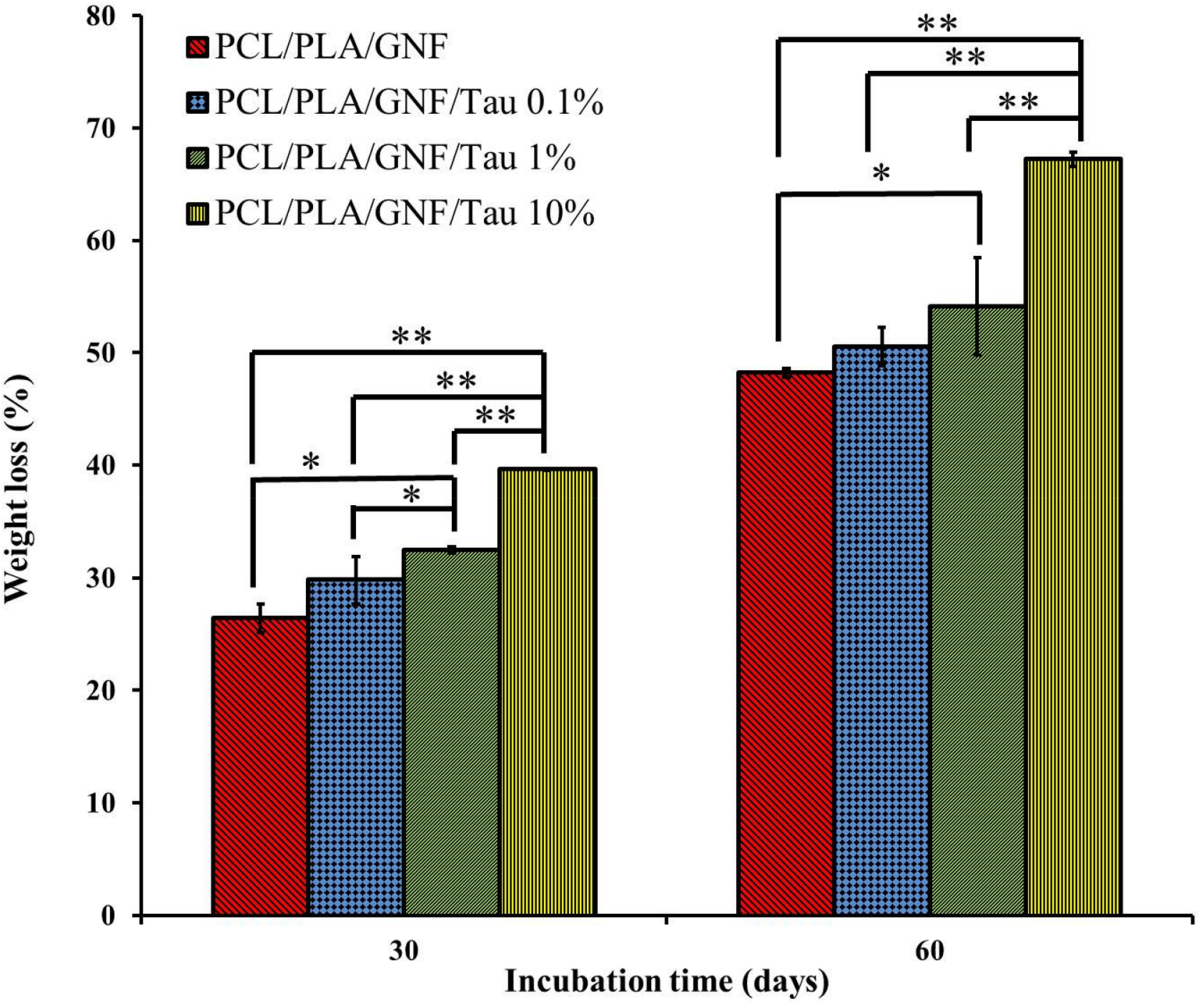
Histogram comparing the prepared scaffolds weight-loss percentages at the end of 60th day post-incubation. Values represent the mean ± SD, n = 3, *p < 0.05 and **p < 0.01 (obtained by Student’s t test).

The implanted scaffolds should be degradable in the body and replaced with the growing bone tissues. Moreover, the rat of the degradation should be in balance with the bone tissue growth, fast degradation left an empty cavity in place, and slow degradation hinderers tissue growth. The weight loss of the prepared scaffolds was evaluated as the biodegradation and the results are presented in Fig. 3.

As shown in Fig. 3, the concentration of Tau had a direct effect on the weight loss of the scaffolds and increasing the concentration of Tau increased the weight loss present of the scaffolds on both 30 and 60 days. The scaffold containing 10% Tau showed the highest weight loss of ≈ 67% on the 60th day. The difference between the scaffolds containing 10% Tau and the other groups was statistically significant (p < 0.01) in both 30th and 60th days. The correlation between hydrophobicity and biodegradability can be attributed to the weight loss obtained results^29^. Since the hydrolysis is the main degradation mechanism of the prepared scaffolds, the incorporation of Tau enhanced weight loss through easier accessibility of water molecules to the polymer changes. Diffusion of the degrading medium into the interior of the scaffolds is the first and determinant step of hydrolysis degradation and increasing the hydrophilicity accelerates the flow of medium into the internal structure of scaffolds^30^.

Park et al.^29^ reported that there is a direct correlation between the hydrophilicity and biodegradability of polyesteramides scaffold. They also suggested that this correlation is through easier accessibility of water molecules to polymer chains. In another study, Oh et al.^31^ fabricated 3D scaffold from PLGA and PLGA/polyvinyl alcohol (PVA) through the melt-molding particulate leaching method. They reported that the hydrophilization of PLGA scaffold by the addition of PVA accelerate the degradation of the scaffolds. Wu et al.^30^ fabricated zein/PCL porous scaffolds by solvent casting–particulate leaching method. They reported that the degradation rate of the PCL scaffold was slower than zein/PCL biocomposite and the rate could be adjusted by varying the amount of zein.

### Mechanical property

**Figure 4:**
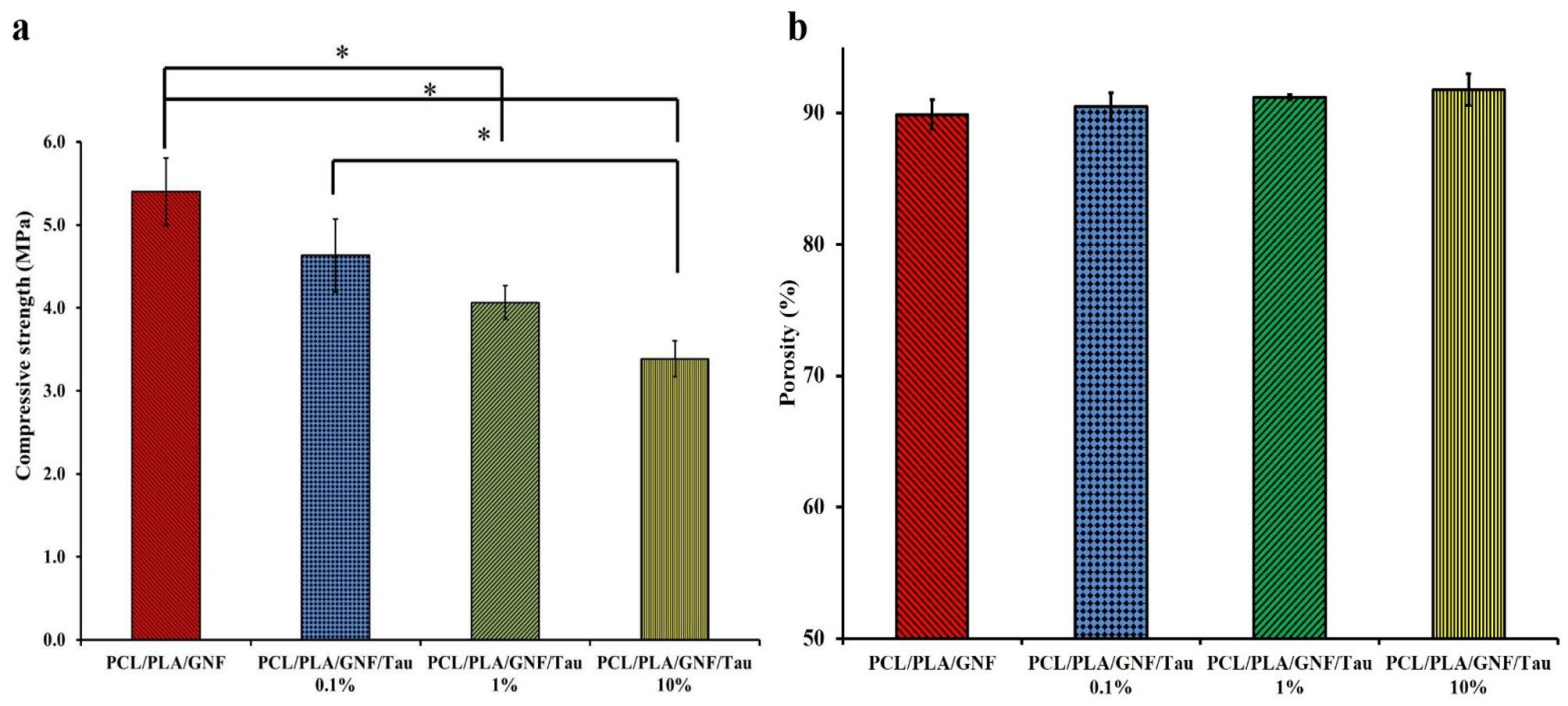
(**a**) The compressive strength obtained from the compression test method, (**b**) the porosity values of the prepared scaffolds obtained with the liquid displacement method. Values represent the mean ± SD, n = 3, *p < 0.05 (obtained by Student’s t test).

A proper bone tissue engineering scaffolds should have acceptable mechanical properties to withstand against the applied stress. The mechanical property of the prepared scaffolds was evaluated using a compression test method, and the obtained compressive strengths were presented in Fig. 4.

As shown in Fig. 4a, the highest compressive strength, 5.4 ± 0.4 MPa, was resulted from PCL/PLA/GNF group and increasing Tau concentration to 10% reduced the compressive strength to 3.3 ± 0.22 MPa. The inverse correlation between the concentration of Tau and the compressive strength can be attributed to the porosity induced by the Tau. The hydrophilic nature of Tau results in more penetration of water molecules and induction of pores with the bigger size and subsequently compromises the mechanical properties^32,33^. Guarino et al.^33^ fabricated 3D scaffolds from poly ε-caprolactone via the phase inversion/salt leaching technique. They reported that there was an inverse correlation between the pore volume fraction and the mechanical strength of the fabricated scaffold.

The functionality of the 3D scaffolds during the tissue engineering applications is substantially related to their porosity and pores size. The porosity of the prepared scaffolds was measured using the liquid displacement method, and the results are presented in Fig. 4b. The results indicated that the porosity value for every scaffold is in the acceptable range and more than 90%. In addition, the incorporation and increasing the concentration of Tau slightly increased porosity percent, while the differences were not statistically significant (p < 0.2). Open porous and interconnected networks are critical for nutrition and waste exchange, cell proliferation and migration for neovascularization and tissue formation. Moreover, the porous scaffolds more effectively mimic the native structure of bone tissue and also induce the formation and release of bioactive molecules such as growth factors, cytokines, and hormones^34–36^.

### Blood compatibility test

**Figure 5:**
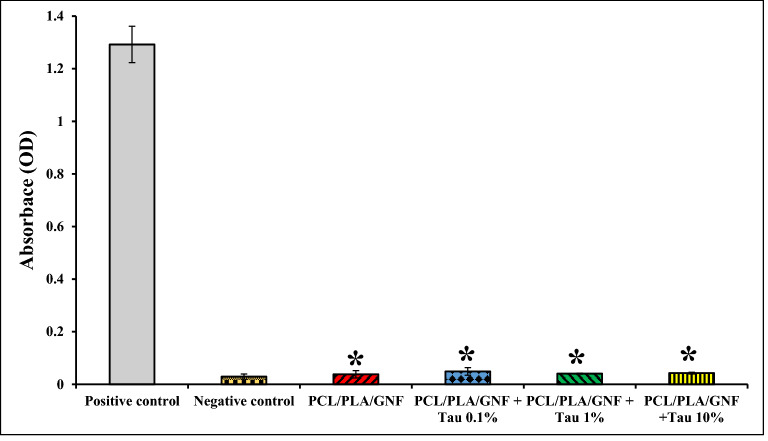
Hemolysis percent of the prepared scaffolds. The negative control was zero. Values represent the mean ± SD, n = 3, *p < 0.05 (obtained by Student’s t test).

Hemocompatibility is the other critical property for an implantable structurethat determinant the interaction of the implanted scaffold with blood cells. The result showed that the hemolysis percent of the prepared scaffolds were lower than the positive control, and the differences are statistically significant (p < 0.05). The results indicated that the prepared scaffolds were hemocompatible and the induced hemolysis values were in the acceptable range (Fig. 5).

### Cytocompatibility and cell proliferation

**Figure 6:**
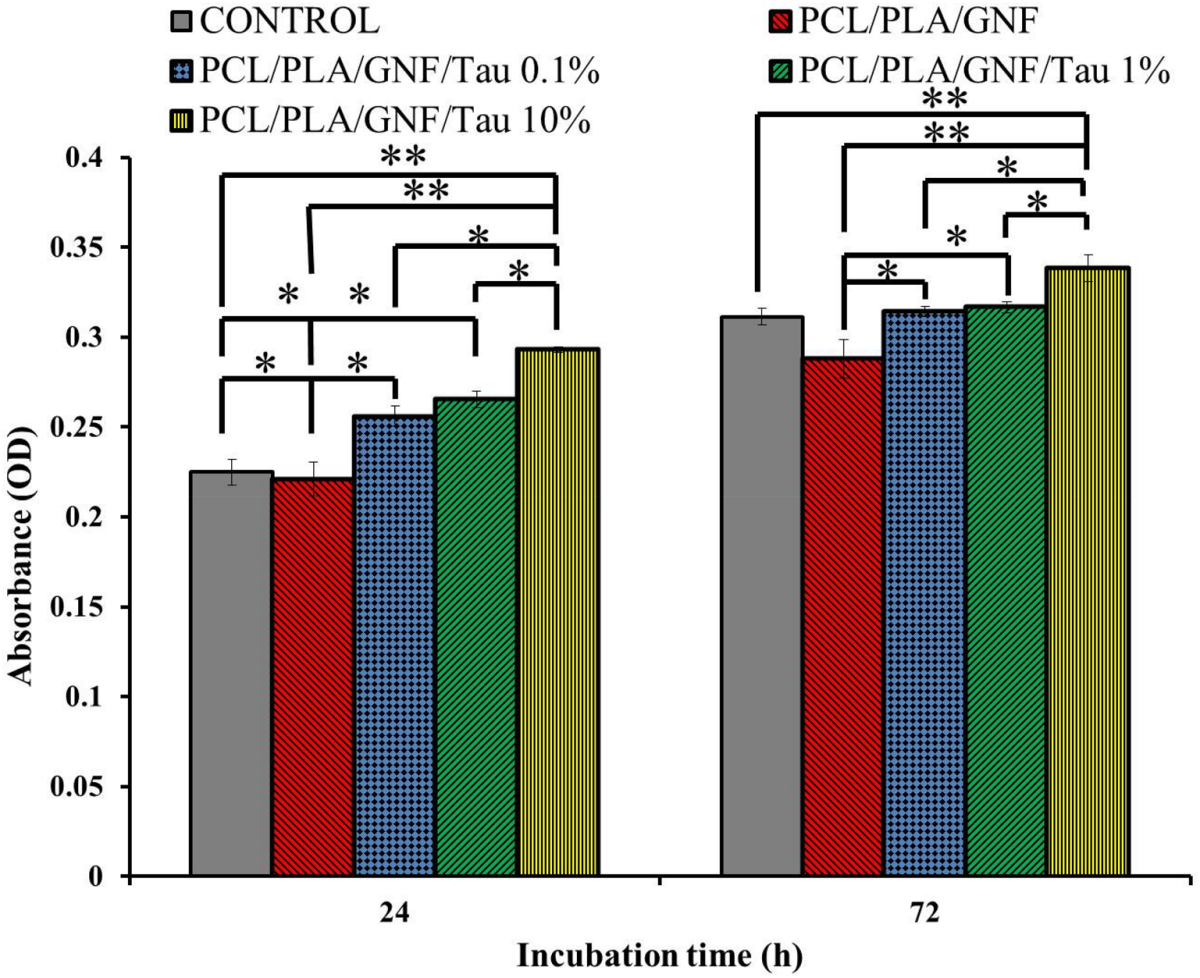
The viability and proliferation of MG-63 on the prepared scaffolds. Values represent the mean ± SD, n = 3, *p < 0.05, **p < 0.01 (obtained by Student’s t test).

The proliferative effects of the prepared scaffolds were evaluated by the MTT assay method, and the obtained results are presented in Fig. 6. MG-63 cells were cultured on the prepared scaffolds, and the proliferation was measured at 24 h and 72 h after cells seeding.

The results showed that the proliferation of the cells on the Tau containing scaffolds was higher than the control (tissue culture plate) and PCL/PLA/GNF scaffold, 24 h after cell seeding. The highest proliferation was achieved with PCL/PLA/GNF/Tau 10%, and the differences were statistically significant (p < 0.01). It is interesting to note that the proliferation of the PCL/PLA/GNF group was lower than the control in both incubation time, and the incorporation of Tau improved the proliferation of the cells. These results indicate that PCL/PLA/GNF/Tau 10% scaffold is suitable for cell growth and proliferation. Our previous study also confirmed that the incorporation of Tau improves the proliferation of cells on the scaffolds^28^.

### In vivo bone formation findings

**Figure 7:**
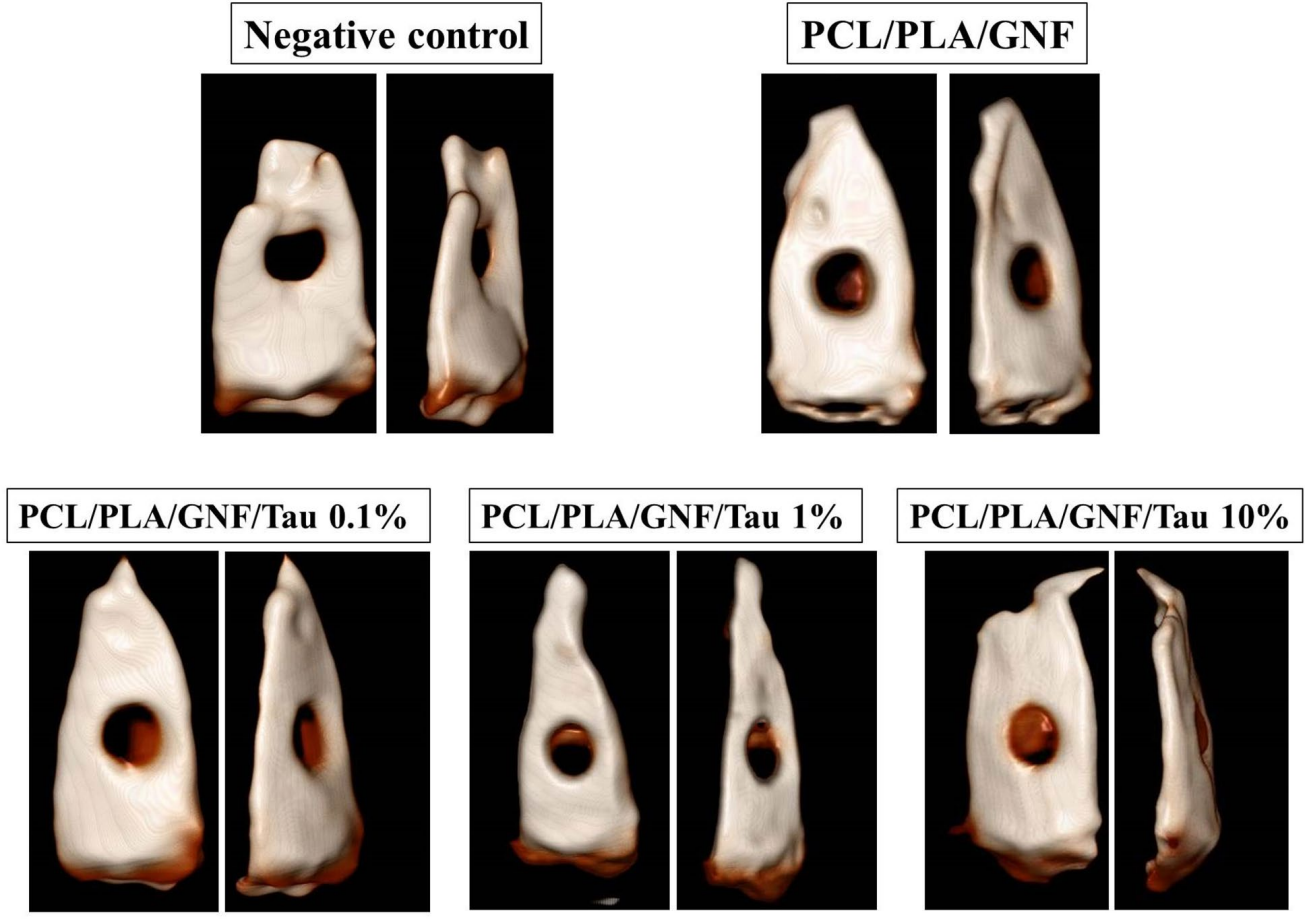
X-ray Computed Tomography (CT) images of damage skull bone. The images were obtained 12 weeks post-implantation.

X-ray Computed Tomography (CT) as the gold standard imaging method on bone was conducted to assess the bone formation within the created fracture under treatment with the fabricated scaffolds. The images were obtained 12 weeks post-implantation of the scaffolds, and the results are presented in Fig. 7.

**Figure 8:**
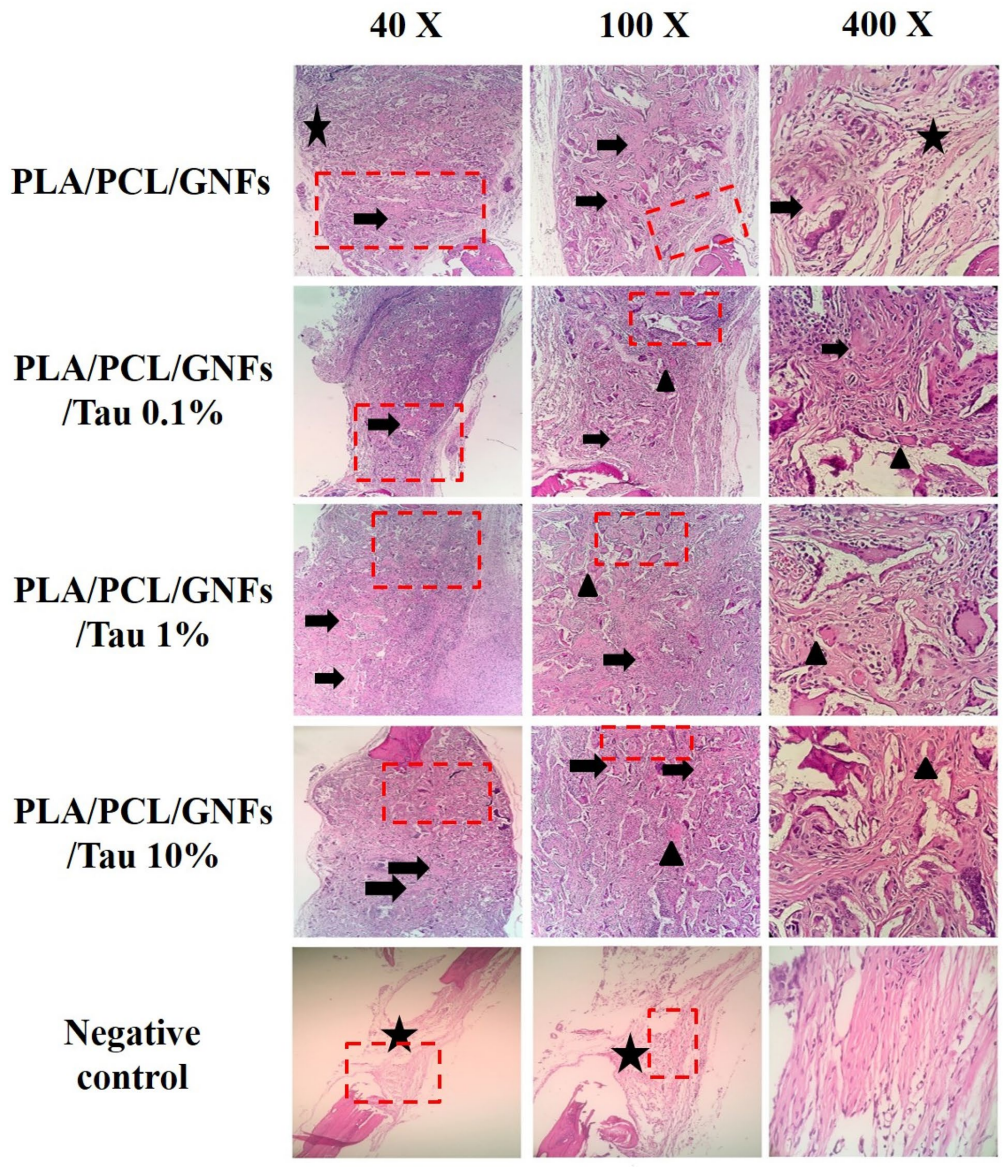
Histopathological sections from the calvarial bone defects treated with the scaffolds. (Stained with H&E). *LACT* Loose areolar connective tissue (star), *NB* new bone formation (thick arrow), *MB* mature bone (arrowhead).

**Figure 9:**
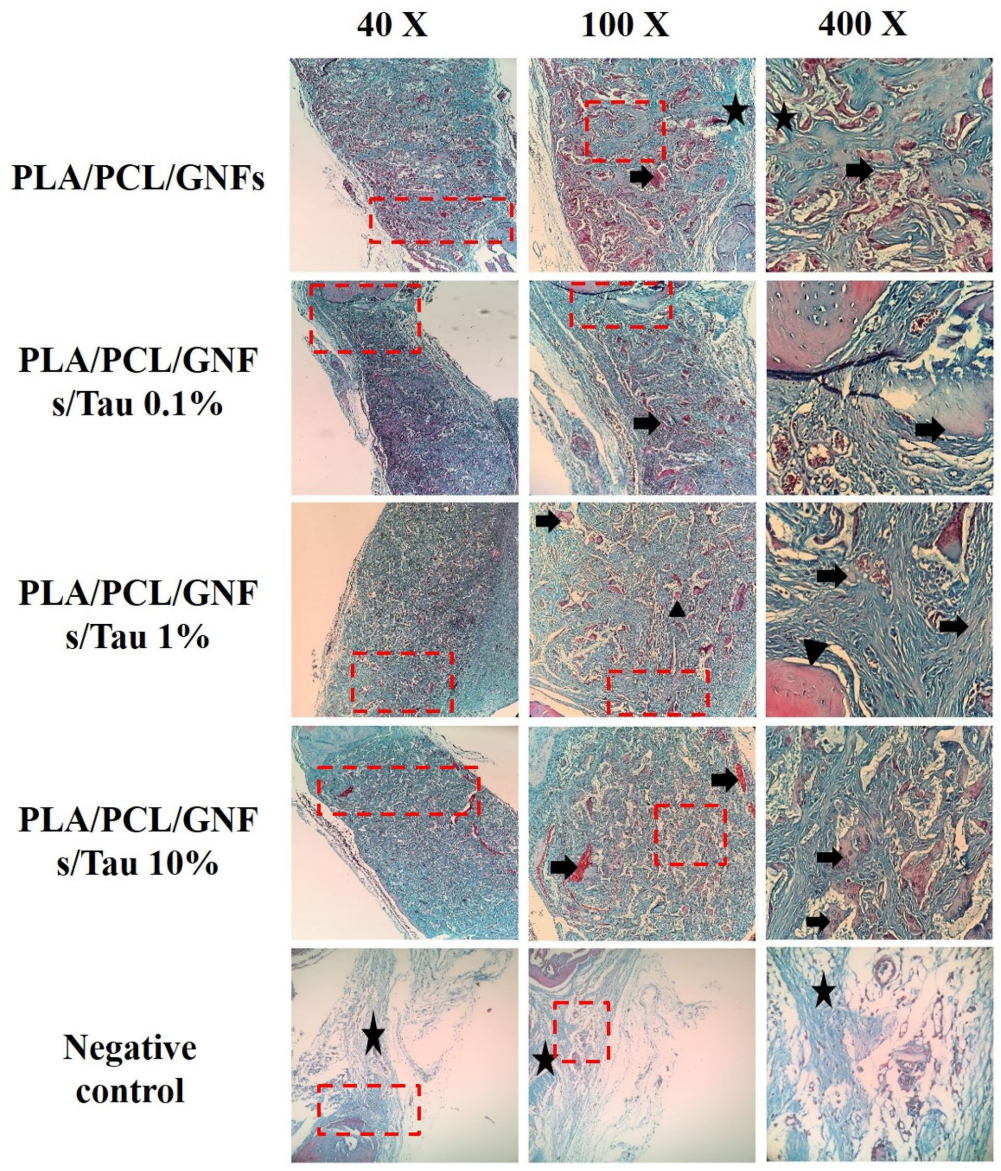
Histopathological sections from the calvarial bone defects treated with the scaffolds. (Stained with TCM). *LACT* loose areolar connective tissue (star), *NB* new bone formation (thick arrow), *MB* mature bone (arrowhead).

As shown in Fig. 7, there are not any signs of new bone formation within the fracture in the negative control group. These images imply that the animal body was not able to heal the damaged bone during 12 weeks. On the other hand, it is apparent that the application of the fabricated scaffolds induced new bone formation. The highest bone formation was obtained with PCL/PLA/GNF/Tau 10%. The histopathological evaluations were conducted using the H&E and MTC staining to further assess the bone healing process in each group (Figs. 8, 9).

As shown in Figs. 8 and 9, the bone defects treated with PCL/PLA/GNF/Tau 1% and PCL/PLA/GNF/Tau 10% scaffold resulted in higher new bone formation compared with the other groups. Moreover, it was observed that in the negative control and PCL/PLA/GNF groups, the defects filled with a loose areolar connective tissue (LACT) (star) that consisted of haphazardly oriented immature collagen fibers, fibroblasts, and newly-formed blood vessels. The results showed that the implanted scaffolds were degraded and almost replaced with the new tissues, including mature bone (MB), and neo-bone (NB). The highest NB, angiogenesis, and woven bone were obtained with treatment by PCL/PLA/GNF/Tau 1% and PCL/PLA/GNF/Tau 10%. While the negative control resulted in the highest LACT, indicating that the defect was not able to heal during 2 months completely by itself.

The positive effect of Tau on bone metabolism and regeneration are reported in other studies. Mi-Ja Choi^37^ showed the beneficial effect of Tau supplementation on femur bone mineral content. In another study, Park et al.^38^ reported collagen synthesis, tyrosine phosphorylation, and alkaline phosphatase activity stimulation in UMR-106 cells under treatment with Tau. They suggested that the observed effect was conducted through the ERK2 signaling pathway. Moreover, Moon et al.^23^ observed that Tau over-expressed Insulin-like growth factor 1 (IGF-1) and serum level of IGF-1 through the phosphorylations of JAK2 and STAT5. They observed that these molecular changes enhance growth plate length, bone volume density (BV/TV), trabecular thickness (Tb.Th), and total porosity. Our findings were in agreement with these conducted studies with the difference in the application route.

## Conclusion

Tissue engineering is an innovative approach toward the regeneration of damaged tissues using the combination of tailored structures as the scaffolds with bioactive substances. The focus of the present study was to develop a functional and bioactive 3D scaffold-based on PCL/PLA containing GNFs and Tau fabricated via the TIPS method. Our results demonstrated that the fabricated scaffolds exhibited bone-regenerating-favorable features such as suitable pore size and interconnected porosity, acceptable hydrophilicity, weight loss, mechanical properties, and hemo- and cytocompatibility. However, the effects of Tau oral administration on bone metabolism and parameters have been evaluated in previous studies, and our study is the first report on the application of a TIPS-based scaffold containing Tau on bone regeneration. Our results revealed that the fabricated PCL/PLA/Gel/Tau 3D scaffold supports bone cell proliferation in vitro and bone regeneration in vivo.

## Materials and methods

### Materials and reagents

Poly (l-lactic) acid (PLA, Mw = 60 kDa), Gelatin powder (bovine skin, type B), poly (ε-caprolactone) [PCL; Mw = 48–90 kDa], Tau ≥ 99%, glutaraldehyde (GA) 25% in H_2_O, ketamine, xylazine, and MTT assay kit were purchased from Sigma-Aldrich (St. Louis, MO). Dimethyl sulfoxide (DMSO), 1,4-dioxane, and acetic acid (AA) were purchased from Merck (Darmstadt, Germany). Dulbecco’s modified Eagle’s medium DMEM, fetal bovine serum (FBS), penicillin and streptomycin were purchased from GIBCO (Grand Island, NY, USA). MG-63 cells and adult male Wistar rats (3 months old, weighing 250–270 g) were obtained from Pasteur Institute, Tehran, Iran.

### Synthesis of electrospun GNFs

A commercial electrospinning device (Fanavaran-nano-meghyas Ltd co Tehran Iran) was used to fabricate GNFs based on our previous study^39^. Briefly, based on our previous study^40^, a 40% (w/v) concentration gelatin solution was obtained by dissolving gelatin powder in AA aqueous [75% (v/v)] under stirrer at room temperature. The applied voltage of 20 kV, feeding rate of 0.40 ml/h, and nozzle to collector distance of 15 cm were chosen as the electrospinning parameters. The fabricated nanofibrous mat was cross-linked with GA 1% for 6 h and then washed by DW several times. The residual GA was neutralized using glycine solution. The neutralized GNFs was transferred to a nitrogen tank and after 24 h crushed to small pieces (GNFs).

### Fabrication of PCL/PLA/GNFs scaffold containing Tau using thermally induced phase separation technique

According to our previous studies^41,42^, PLA and PCL with the mass ratio of 1:1 were dissolved in 1,4-dioxane to form a 10% (w/v) solution. Then GNF (10% w/w) was added to 10 ml of the prepared PCL/PLA solution with 40% weight of PCL/PLA. Tau was added to the prepared solution and stirred at room temperature for 12 h to obtain the final concentrations of 0.1%, 1%, and 10% of Tau in PCL/PLA/GNF solution. The mixtures were frozen at − 80 °C for 24 h and freeze-dried (Telstar, Terrassa, Spain) for 48 h, and the dried scaffolds were obtained after freeze-drying.

### Characterization of the scaffolds

#### Surface morphology

The surface morphology of the prepared scaffolds was observed by using a Scanning Electron Microscope (SEM; Philips XL-30) at the accelerating voltage of 15 kV. The SEM images were obtained from the cross-section of the scaffolds after freeze-drying and sputter coating with a thin layer of gold.

#### Contact angle measurement

The wettability of the prepared scaffolds was evaluated using a water contact angle measuring system (G10, KRUSS, Germany) in the static mode with the sessile drop method. Five samples were used for each test, and the average value was reported with standard deviation (± SD).

#### Weight loss assessment

The weight loss measurement was carried out according to our previous paper^43^. Briefly, the prepared scaffolds were gently cut into 20 mm diameter disks, weighed, and immersed in the glass test tubes filled with 15 ml of PBS containing 250 ng/ml amphotericin B, 100 unit/ml penicillin, and 100 µg/ml of streptomycin. The samples were maintained at 37 °C in an incubator for 60 days. After the selected time points, the scaffolds were recovered and dried to a constant weight. Equation (1) was used to calculate the weight loss, where “W_0_” is the initial weight of samples and “W_1_” is the dry weight after removing from the media:

1 $$Weight\;loss\;\left(\%\right)=\frac{W0-W1}{W0}\times 100$$

#### Mechanical properties measurement

The compression test method was applied to evaluate the mechanical properties of the scaffolds^43^. Three dried cylindrical samples of each scaffold (height and diameter of 20 mm and 10 mm, respectively) were prepared and evaluated using a mechanical testing machine (Santam, Karaj, Iran) at a crosshead speed of 0.5 mm/min. The apparatus was equipped to a 1 kN load cell.

### Porosity assessment

The liquid displacement method was used to determine the porosity of the prepared scaffolds based on Eq. (2)^39^

2 $$ Porosity \; \left( \%  \right) = \frac{{V_{1}  - V_{3} }}{{V_{2}  - V_{3} }} \times 100 $$

where V_1_ is the initial volume of absolute ethanol, V_2_ is its volume after scaffold soaking (and ethanol filled the pores), and V_3_ is the volume of the ethanol after the scaffold removal.

### Blood compatibility evaluation

The hemocompatibility test was carried out on human fresh anticoagulated blood collected form volunteers. The study was approved by the Ethics Committee in Kermanshah University of Medical Sciences (Ethics board approval number: IR.KUMS.REC.1397.545). The methods in the study were in accordance with the guidelines of the Declaration of Helsinki. Written informed consent was obtained from all subjects before blood donation. The Each scaffold was incubated with 0.2 ml of the blood diluted with normal saline for 60 min at 37 °C. After passing the incubation time, the incubated blood was centrifuged at 1,500 rpm for 10 min and the resulted supernatant transferred to a 96-well plate, and the absorbance was measured at 545 nm by utilizing the Anthos 2020 (Biochrom, Berlin, Germany) microplate reader. The absorbance was attributed to the hemoglobin leaked from red blood cells (RBC) and hemolysis degree was calculated using Eq. (3)^43^:

3 $$Hemolysis \; \%=\frac{Dt - Dnc }{Dpc - Dnc} \times 100$$

where D_t_, D_nc,_ and D_pc_ are the absorbance of the sample, the absorbance of the negative control, and the absorbance of the positive control. The negative and positive controls were 0.2 ml of blood diluted with normal saline and deionized water, respectively.

### Cell viability and proliferation assessment

MTT assay was used to quantitatively measure the viability and proliferation of MG-63 cells cultured on the prepared scaffolds. The samples were punched circulatory and put at the bottom of the 96-well plate and sterilized with UV light 15 min. MG-63 cells at a density of 7 × 10^3^ cells/sample were seeded onto the samples and incubated for 1 and 3 days under standard culturing conditions. At each time point: the culture media was depleted, cells were washed with sterile PBS three times, and 150 µl of new DMEM containing 20 µl of 5 mg/ml MTT solution was added to each well followed by incubation for 4 h. After passing this incubation time and formation of purple formazan crystals, the media was replaced with 100 µl DMSO to dissolve the formazan crystals. Then, 100 μl aliquots were transferred to a 96-well plate, with three replicates per sample and the absorption was read at 570 nm using a microplate reader.

### In vivo studies

The healing efficacy of the prepared scaffolds was examined in an animal model. The animal studies were conducted based on the ethical committee of Kermanshah University of Medical Sciences and were carried out in accordance with the university guidelines. Thirty healthy adult male Wistar rats were randomly divided into five groups (six rats per group). The groups include (1) PCL/PLA/GNF scaffold without Tau, (2) PCL/PLA/GNF/0.1% Tau scaffold, (3) PCL/PLA/GNF/1% Tau scaffold, (4) PCL/PLA/GNF/10% Tau scaffold, and (5) Negative control group. The animals were anesthetized by intraperitoneal injection of Ketamine 100 mg/Xylazine 10 mg/kg bodyweight. A trephine (Meisinger) with an external diameter of 7 mm and an internal diameter of 6 mm at a speed rate of 1,000 rpm was used to make a 7 mm spherical incision in the calvaria (skull) of the animals. Subsequently, the prepared scaffolds were put into the defects and the periosteum was repositioned and closed with No. 6.0 nylon suture (SUPA medical devices, Tehran, Iran). No. 3.0 nylon suture (SUPA medical devices, Tehran, Iran) was used to close the skin.

### 3D-computed tomography (CT) imaging

Somatom Emotion CT scanning system (Siemens, Erlangen, Germany) was used to visualize bone formation at the end of 12th-week post-operation.

### Histological analysis

The histological analysis was conducted according to our previous study^41^. Briefly, the animals were euthanized 12th-week post-operationand the harvested tissues were fixed in neutral buffered formalin (NBF, 10%; pH 7.26) for 48 h. The treated bones were decalcified by storage in nitric acid (5% v/v) for 10 days. The decalcified bones then went through histological fixation and dehydration processes, embedded in paraffin. Sections (5 µm thick) were cut and stained with Haematoxylin and Eosin (H&E) and Masson's trichrome (MCT). The resulted histological slides were evaluated by the independent pathologist, using light microscopy (Olympus BX51; Olympus, Tokyo, Japan). The amount of the newly formed cartilage or bone was assessed as well as the amount of the remaining implants in the total area of the section. Furthermore, the histomorphometric analysis was carried out and the appearance and number of different cells including; fibroblasts, fibrocytes, chondroblasts, chondrocytes, osteoblasts, osteocytes, giant cells, macrophages, lymphocytes, neutrophils, as well as other constituents such as blood vessels, and new cartilage and bone tissues were studied. The resulted data were analyzed using Image-Pro Plus (version 6, Media Cybernetics, Inc., https://www.mediacy.com/imageproplus, Silver Spring, USA).

### Statistical analysis

The results were statistically analyzed by Minitab (version 17, Minitab Inc., https://www.minitab.com/en-us/, State College, USA) software using Student’s t-test and the data were expressed as the mean ± standard deviation (SD)^43^. In all evaluations, p < 0.05 was considered as statistically significant.

### Ethical approval

The study was approved by the Ethics Committee in Kermanshah University of Medical Sciences (Ethics board approval number: IR.KUMS.REC.1397.545). The methods in the study were in accordance with the guidelines of the Declaration of Helsinki. Written informed consent was obtained from all subjects before blood donation. The animal study was conducted on 30 male adult Wistar rats after approval of the ethics committee of Kermanshah University of Medical Sciences (Ethics board approval number: IR.KUMS.REC.1397.545). All applicable international, national and institutional guidelines for the care and use of animals were followed.

## Data Availability

The datasets generated during and/or analysed during the current study are available from the corresponding author on reasonable request.
